# Growth Hormone Resistance—Special Focus on Inflammatory Bowel Disease

**DOI:** 10.3390/ijms18051019

**Published:** 2017-05-09

**Authors:** Christoffer Soendergaard, Jonathan A. Young, John J. Kopchick

**Affiliations:** 1 Novo Nordisk A/S, Global Research, Maaloev 2760, Denmark; c.sondergaard@gmail.com; 2 Edison Biotechnology Institute, Ohio University, Athens, OH 45701, USA; jy811107@ohio.edu; 3 Department of Biological Sciences, Ohio University, Athens, OH 45701, USA; 4 Department of Biomedical Sciences, Heritage College of Osteopathic Medicine, Ohio University, Athens, OH 45701, USA

**Keywords:** growth hormone, IL-1β, IL-6, inflammation, inflammatory bowel disease (IBD), intestine, resistance, STAT5, TNF-α

## Abstract

Growth hormone (GH) plays major anabolic and catabolic roles in the body and is important for regulating several aspects of growth. During an inflammatory process, cells may develop a state of GH resistance during which their response to GH stimulation is limited. In this review, we will emphasize specific mechanisms governing the formation of GH resistance in the active phase of inflammatory bowel disease. The specific molecular effects mediated through individual inflammatory mediators and processes will be highlighted to provide an overview of the transcriptional, translational and post-translational inflammation-mediated impacts on the GH receptor (GHR) along with the impacts on GH-induced intracellular signaling. We also will review GH’s effects on mucosal healing and immune cells in the context of experimental colitis, human inflammatory bowel disease and in patients with short bowel syndrome.

## 1. Introduction

Growth Hormone (GH) is a 191 amino acid protein that is secreted primarily by somatotrophic cells of the anterior pituitary. GH potentiates anabolic action on muscle and bone and catabolic action on adipose tissue. Additionally, GH promotes fluid retention via the kidney, and metabolic effects on the liver. Some, but not all, of these effects are brought about by the action of Insulin-like growth factor (IGF)-1 which is produced in response to GH in most tissues with the liver predominating. Thus, GH has both direct and indirect physiological effects [1]. These dual effects have been nicely presented in terms of mouse growth. In this study, GH is reported to be responsible for 14% of mouse growth with IGF contributing 35% and a combination of GH & IGF-1 adding 34% [2]. The salient point is that GH and IGF-1 have both independent as well as overlapping functions in terms of their physiological actions.

**Figure 1:**
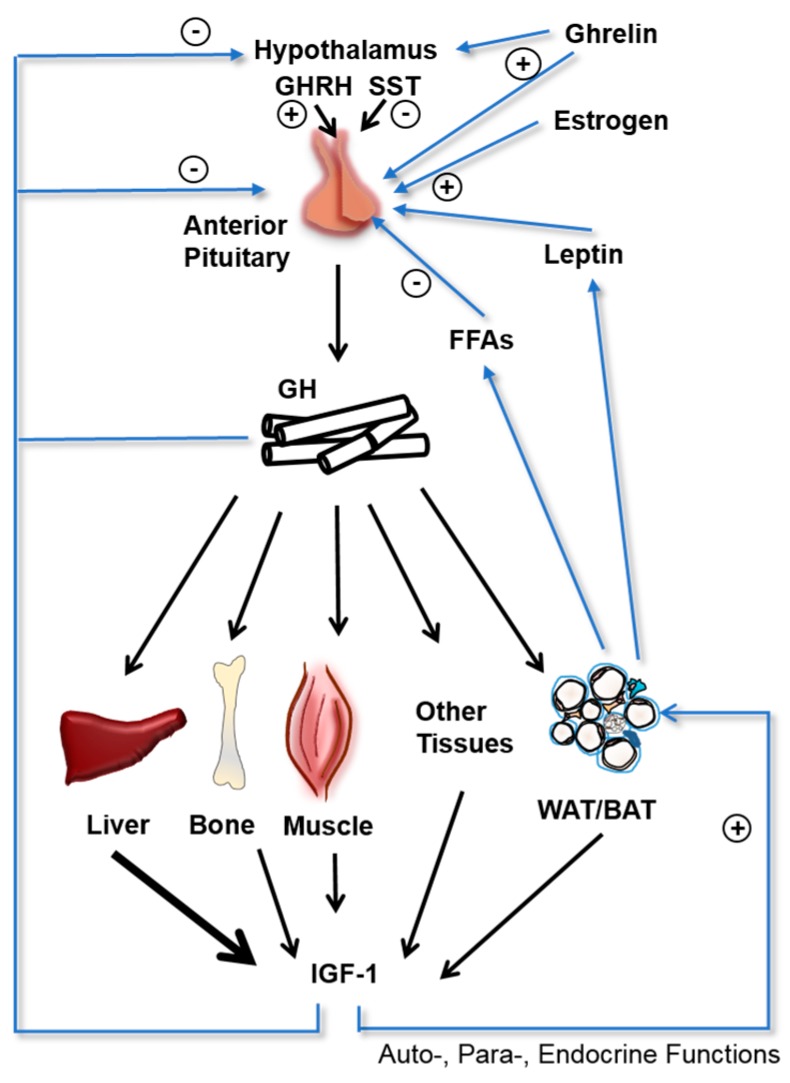
Regulation of growth hormone (GH) and Insulin-like growth factor (IGF)-1 Secretion. GH secretion by the anterior pituitary into the circulation is regulated in a complex manner. The hypothalamus secretes GH releasing hormone (GHRH) and Somatostatin (SST) which increase and decrease GH secretion, respectively. Endocrine GH travels through the bloodstream and stimulates IGF-1 secretion in multiple target tissues, with the liver as the primary source of circulating IGF-1. GH secretion is regulated by multiple negative feedback loops, with high GH levels and IGF-1 levels inhibiting GH secretion at the hypothalamic and pituitary level. GH secretion is also negatively regulated by serum free fatty acids (FFAs) and positively regulated by ghrelin, estrogen, and leptin. Black arrows indicate actions of GH on target tissues and resulting IGF-1 generation with the liver being the dominant source. Blue arrows indicate positive (+) and negative (−) regulatory mechanisms on GH secretion.

GH secretion by the somatrophs of the pituitary gland is complex, involving signals derived from the hypothalamus, pituitary, liver, and stomach (Figure 1). Different subsets of hypothalamic neurons secrete GH releasing hormone (GHRH) and somatostatin, which stimulate and inhibit GH secretion from the pituitary, respectively. GH secretion is also regulated by multiple feedback loops. High serum GH levels inhibit GH release by the pituitary and GHRH release by the hypothalamus, and stimulate somatostatin release by the hypothalamus. Increased serum IGF-1 levels also inhibit GH release by the pituitary. Ghrelin, an orexigenic peptide hormone secreted primarily by the stomach, also increases GH secretion by the pituitary, linking gastrointestinal function to GH [3].

GH-induced intracellular signaling is mediated by the GH receptor (GHR) that is expressed by most cells throughout the body. GHR is a class I cytokine receptor and consists of 3 domains: an extracellular domain (the site of GH binding), a transmembrane domain, and an intracellular domain, which is responsible for the initiation of GH induced intracellular signal transduction. GHR exists on the cell membrane as a preformed homo-dimer. Two distinct sites on GH (Sites 1 and 2) must bind to two distinct sites on the GHR dimer that promotes a rotation of the GHRs, ultimately activating Janus Kinase 2 (JAK2), thus initiating the intracellular signaling cascade [4]. Also, a subset of the GHR homodimer can be cleaved at the cell membrane releasing the extracellular domain (known as the GH binding protein (GHBP)) into the circulation [5].

**Figure 2:**
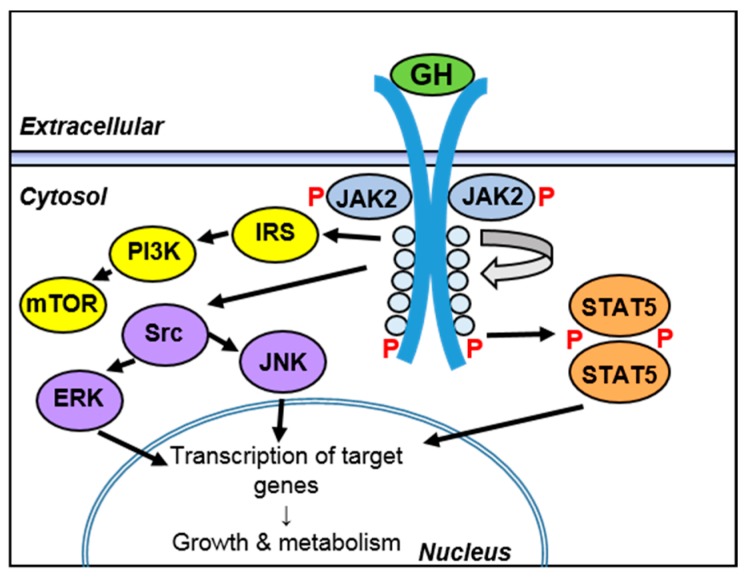
Intracellular GH signaling. GH binds to a pre-formed GHR dimer on the cell surface, leading to a conformational change in the intracellular domain that results in phosphorylation (P) and activation of signal transducer and activator of transcription (STAT)5 through Janus Kinase (JAK)2. Phosphorylated (active) STAT5 then travels to the nucleus and regulates the transcription of GH target genes. GHR can also signal through the mTOR pathway through insulin receptor substrate (IRS) and phosphoinositide-3 kinase (PI3K), and can activate c-Jun N-terminal kinase (JNK) and extracellular signaling regulated kinase (ERK) through activation of Src kinase. Arrows show activation sequence and nuclear translocation downstream of GHR.

As stated above, GH-induced intracellular signaling is mediated by the intracellular domain of GHR. JAK2 is bound to the intracellular domain of each of the GHR monomers. Upon GH binding, a conformational change or rotation of the GHR occurs that ultimately exposes the kinase domains of the JAK2 proteins, which allows them to self-activate and phosphorylate GHR, providing docking sites for signal transducer and activator of transcription 5 (STAT5). Subsequently, a tyrosine residue on these “docked” STAT5 molecules becomes phosphorylated via activated JAK2. This promotes de-docking and dimerization of the phosphorylated STAT5, which ultimately enters the nucleus and participates in regulation of GH-induced genes. Additionally, GHR may signal through other pathways, including mTOR (through insulin receptor substrate (IRS) and phosphoinositide-3 kinase (PI3K)) and c-Jun N-terminal kinase (JNK) and extracellular signaling regulated kinase (ERK) through mitogen-activated protein kinase (MAPK) [4,6] (Figure 2).

As stated above, although many of the anabolic effects of GH are mediated through stimulation of IGF-1 released by hepatocytes, GH also has direct effects on other tissues. One effect is the extrahepatic production of IGF-1 which can act as a paracrine/autocrine signal in target tissues [7]. Besides the previously stated diverse effects of GH on target tissues which include promotion of lipolysis in adipose tissue, nutrient uptake in muscle tissue, and gluconeogenesis and glycogenolysis in the liver, GH also has diabetogenic (anti-insulin) activity, one of the first reported effects of GH [8,9].

## 2. Inflammatory Bowel Diseases

The inflammatory bowel diseases (IBD) include the two prevailing entities: Crohn’s disease (CD) and Ulcerative colitis (UC). These are chronic conditions and are associated with an intermittent course of active inflammation.

Clinically, the two conditions present themselves with diarrhea, bloody stools and abdominal discomfort, and CD is additionally associated with malnutrition. Biochemically, active IBD is associated with upregulation of several pro-inflammatory mediators locally in the affected tissue, including tumor necrosis factor (TNF)-α, interleukin (IL)-1β, and IL-6, and in more severe cases, systemic levels of these mediators have been found to be significantly elevated including C-Reactive Protein (CRP) [10,11,12]. The etiology of IBD is incompletely understood, however, environmental factors, genetic predisposition and interaction with the commensal microbiota are known to play major roles in propagation of the inflammatory process [13,14].

The integrity of the mucosal barrier is essential to safeguard intestinal homeostasis. This barrier becomes impaired in patients with active IBD, where several aspects of the barrier function are affected including release of anti-microbial peptides, mucin secretion, and expression and assembly of tight-junction proteins [15,16,17,18]. Malnutrition, resulting from systemic inflammation and a local impact on absorption by the affected epithelium, may lead to general growth retardation among children with IBD and may also impact the ability to circumvent the ongoing inflammatory process. Malnutrition is a concern among IBD patients, where the prevalence of under nutrition and severe under nutrition was estimated to 25.0–69.7% and 1.3–31.6%, respectively [19]. The condition is most pronounced among patients with CD with small intestinal affection. Weight loss combined with prolonged disease activity increases the risk for developing malnutrition [20,21].

Early studies have shown that patients with active CD have altered metabolic profiles and show a hyper catabolic state [22,23]. A study conducted in the early 1980s found that 23% of pediatric cases of IBD showed growth retardation, which was twice as frequent for children with CD [24]. Later, additional studies have shown growth retardation to be most frequent in CD with a frequency of around 10–19% and to rarely manifest in children with UC [25,26,27]. The cause of growth retardation during CD is likely a combination of impaired nutritional uptake and a direct effect of inflammation on the GH-IGF-1 axis resulting in reduced levels of bioavailable IGF-1 [27,28].

In terms of treatment of IBD, restitution of the mucosal barrier, or “mucosal healing”, is considered the main success criteria as it is the best prognostic marker for the induction of longer-lasting remission [29]. Treatment of mild cases of IBD consists of oral or topical immunomodulators, while longer-standing or more severe cases are supplemented with steroids. In steroid-refractory patients, treatment using neutralizing antibodies against TNF-α, leukocyte integrins, or cytokine receptors may be employed [30,31].

In cases of unsuccessful treatment, patients may ultimately undergo surgery to remove the affected bowel segments or the entire large bowel in the case of UC. Surgery becomes the consequence for approximately 47% and 16% for CD and UC 10 years after diagnosis, respectively [32].

Resection of either the large bowel or extended parts of the small intestine may lead to development of short bowel syndrome (SBS) a condition associated poor absorption of nutrients. Depending on the resected area, the absorption of water, minerals, vitamins, fat, protein, or other nutrients may be compromised [33].

This review will focus on the molecular mechanisms influencing and regulating the cellular responsiveness to GH. This includes the molecular mechanisms involved in the formation of GH resistance. The potential beneficial effects of GH on selected areas of IBD-mediated inflammatory processes will be reviewed and addressed in perspective with previous experimental colitis in animals and human clinical data.

## 3. Mechanisms of Inflammation-Driven Growth Hormone (GH) Resistance

The responsiveness of GH may be impacted in two basic ways: by regulation of the level of GHR, and by regulation of GH-induced intracellular signaling pathways. Most studies have investigated the mechanisms underlying GH resistance in hepatocytes using GH-induced IGF-1 response as the readout. The inflammatory impact on extrahepatic cells are likely similar, although few reports exists.

### 3.1. Regulation of GH Receptor (GHR) Expression

**Figure 3:**
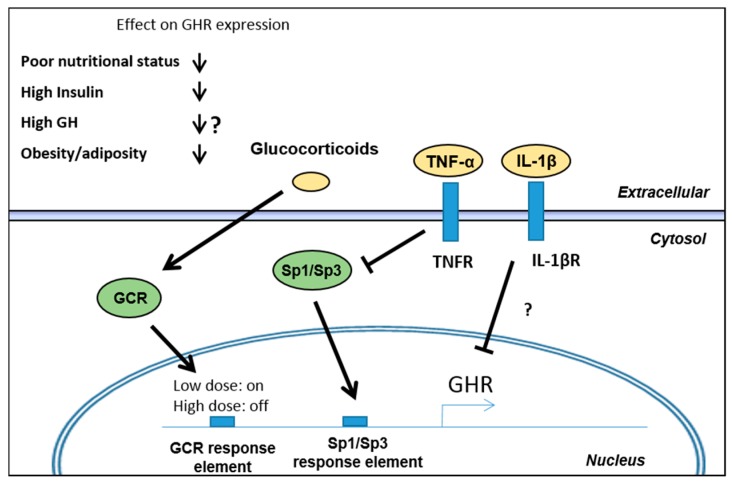
Regulation of GHR expression. Multiple metabolic and inflammatory factors are involved in the regulation of GHR transcription, mediated through both known and unknown mechanisms. Metabolic parameters including poor nutritional status, high insulin levels and obesity negatively impacts GHR expression. The effect of GH on GHR expression is imperfectly understood as applied concentrations, dosing regimen, e.g., pulse-like or chronic, as well as the model systems used show conflicting results. Arrows and blocked arrows indicate suggested effects by glucocorticoids, TNF-α and IL-1β through their respective receptors on GHR transcription. “?” means unknown mode of action

As GH plays a role as a metabolic regulator, regulation of GHR expression is also controlled by factors including nutritional status, GH, insulin, and obesity/adiposity (Figure 3) [34,35,36,37].

The metabolic state and the availability of external nutrients play a role in regulating GHR expression. Fasting rats show reduced expression of both GHR and IGF-1 receptor, which correlates with the duration of fasting [38,39]. In patients having anorexia nervosa, similar resistance to GH has been described, which is characterized by elevated GH and decreased circulating IGF-1 [34,40]. Accordingly, the effects of malnutrition, as observed during severe cases of IBD or SBS, potentially impacts GHR expression negatively. The mechanisms underlying the impact of nutrition on GHR expression and sensitivity are not completely understood. It has been suggested that complete fasting has a higher impact on GHR expression than caloric or protein restriction alone and that re-supplementation leads to restoration of the GHR expression in rats [38,41,42,43,44]. Species-specific differences have been observed in relation to the effect of food restriction on GH-secretion. It has been shown that rats respond to energy restriction with a decrease in GH secretion [45], whereas humans show increased GH secretion [46,47,48]. This indicates that factors downstream of GH secretion are regulating GH sensitivity in humans and that caution should be taken when comparing animal studies. Besides anorectic individuals, pure nutritional deficiency is rare in the developed world and is often connected with underlying conditions like systemic inflammation or celiac disease, where energy expenditure is increased and nutritional uptake is impaired. A recent study identified Sirtuin 1 (SIRT1) as an important fasting-induced regulator of GH sensitivity in the liver [49]. SIRT1 is an intracellular deacetylase whose expression is enhanced during fasting. SIRT1 deacetylates STAT5 resulting in an inability of STAT5 to dock to the activated GHR hence limiting GH signaling. In a similar way fasting-induced FGF-21 has been also shown to reduce the hepatic STAT5 level which likewise limits GH responsiveness [50]. Conclusively, multiple energy-sensing pathways apparently regulate GH response in order to control energy expenditure and maintain euglycemia [51].

Insulin has also been shown to directly impact both the expression and the protein level of GHR although the mechanism is complex. Early studies have shown that type I diabetic patients are in a state of GH resistance [52,53]. At the same time, it has been shown that female diabetic rats (streptozotocin-induced) have reduced GH binding due to a reduction in binding sites which was increased again following insulin supplementation [54]. In vitro, the human hepatoma cell line HuH7 also respond to insulin (10 nM) by up-regulation of GHR, although the study focused on concentrations of insulin superseding physiological levels [55]. To complicate the subject, more recent studies have indicated that high levels of insulin (10 nM) negatively impacts GHR expression in rat hepatocytes (H4IIE). This is possibly mediated by insulin signaling through the PI3K and MAPK/ERK pathways which leads to reduced GHR mRNA transcription as well as GHR protein levels [56,57]. This finding has a potential impact on type 2 diabetic individuals having increased insulin over extended periods of time, although diabetes associated factors like increased free fatty acid (FFA) levels, decreased ghrelin and low-grade inflammation in this patient group also have the potential to impact GHR expression [35,58,59]. Conclusively, the impact of insulin on GHR has been inconsistently reported, although the studies collectively show that deregulated insulin, high (e.g., type II diabetes) or low (malnutrition), negatively impacts hepatic GHR levels. Additional clarity of this subject in a human setting is, however, still needed.

Supraphysiological levels of GH in cell cultures (human glomerular mesangial cell line, mouse fibroblasts, human IM-9 lymphocytes, and human T-47D mammary gland epithelium) leads to decreased GHR expression, which might function as a compensatory mechanism for the cells to reduce the level of GH action. On the other hand, rat epiphyseal chondrocytes increased GHR expression following GH stimulation. The effects of GH on GHR expression is, accordingly, inconsistently reported and may depend on the type of exposure; cell/tissue type, dosages and dosage regimens e.g., pulse-like or chronic stimulation [36,60,61].

Besides these basic metabolic regulatory mechanisms and the effects of GH itself, the presence of inflammatory mediators also impacts the level of functional surface exposed GHR. A series of studies have shown an effect on GH-induced signaling by some of the key inflammatory mediators in IBD including TNF-α, IL-1β, and IL-6 as described below. These mediators are able to impact either the expression of the GHR or the downstream signaling intermediates. Most studies have focused on cultured hepatocytes or rodent and quantify IGF-1 expression or synthesis as a surrogate marker of GH activity. Numerous studies have pointed to direct effects of TNF-α and IL-1β in inhibiting GHR expression [62,63,64,65,66], an effect which is enhanced by simultaneous stimulation with both [65]. These effects were attenuated when antagonists of TNF-α or IL-1β were used or by genetic ablation of their cognate receptors [62,63]. The molecular mechanisms governing inflammation-induced GHR expressional changes are incompletely understood. However, a direct effect of TNF-α on the GHR promoter/enhancer elements have been described in murine BNL CL.2 hepatocytes. Here, TNF-α reduced DNA binding of the transcription factors SP1 and SP3 to the GHR promoter/enhancer leading to repression of transcription [63]. In IL-10 gene disrupted mice, experimental colitis is also characterized by reduced GHR expression in the liver driven by TNF-α-induced reduction in nuclear SP3. Blockade of TNF-α restored GHR expression and STAT5 activation in these animals [63,67]. A similar effect was observed in human HEK293 cells and in mature human SGBS adipocytes, where TNF-α along with HIF-1α and glucocorticoids affected GHR expression through specific response elements at the GHR promoter/enhancer region [68]. Accordingly, the same authors also observed reduced GHR expression in adipocytes from obese individuals, which possibly coincide with local low-grade obesity-related inflammation [35].

The mechanisms by which IL-1β impacts GHR expression is less studied, and reports are conflicting. They show either a direct effect of IL-1β on GHR expression [65] or an IL-1β dependent decrease in IGF-1 and SPI1 expression, through an unidentified mechanism [69]. A synergistic additive effect of TNF-α and IL-1β on repressing GHR expression has been shown in Huh-7 human hepatoma cells [65]. A general and additive effect of TNF-α and IL-1β on rat metatarsal bone growth also points to an inflammatory impact on growth, although the authors did not quantify GHR expression or subsequent down-stream signaling [70]. In vivo treatment of colitis in IL-10 knock out mice using anti-TNF-α antibodies increased GH action in terms of STAT5 phosphorylation as well as GHR expression levels in the colon [71] and in clinical evaluations anti-TNF-α treatment increases systemic IGF-1- and IGF binding protein-3 levels in IBD patients [72,73]. To our knowledge no reports exist on the effect of anti-TNF-α on GHR expression in humans.

The direct effects of another major inflammatory mediator, IL-6, seem to act independent of regulation of GHR expression. Instead IL-6 impacts the expression of regulatory molecules of the suppressor of cytokine signaling (SOCS)-family as detailed in the next section.

Besides treatment with biologics, e.g., antibodies targeting cytokines or their receptors, a first line of treatment for patients with IBD is glucocorticoids or steroid hormones along with immunomodulators like thiopurines. Importantly, long-term treatment with steroids can lead to growth defects in children and impact the GH-IGF-1 axis negatively through an impact on GH secretion and GH responsiveness [74]. Erman A. et al. have shown that the synthetic glucocorticoid (dexamethasone) can lead to interaction between the glucocorticoid receptor and putative glucocorticoid response element at the GHR promoter/enhancer in HEK293 and SGBS cells [68]. This interaction induced a biphasic effect where low dose dexamethasone enhances the GHR expression, while high dose leads to alleviation of this effect with a return to normal levels. In relation to glucocorticoid treatment in IBD, this means that high dose exogenous steroids may de-sensitize the positive effects of low levels of endogenous cortisol.

### 3.2. Post-Transcriptional Regulation of GHR Levels

**Figure 4:**
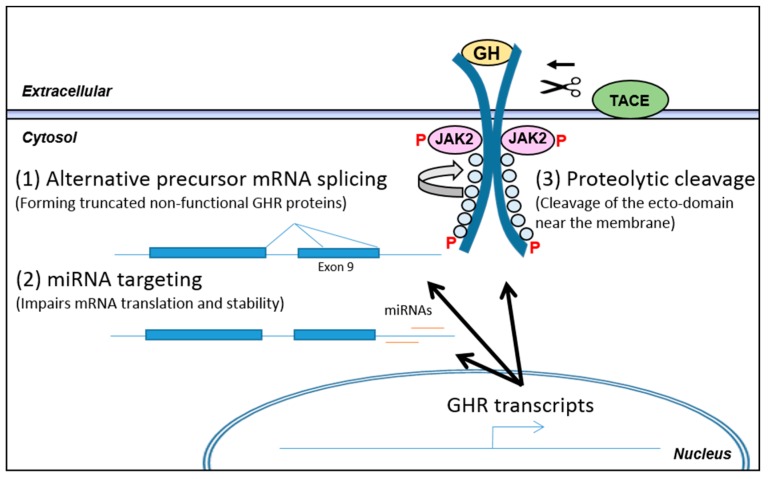
Post-transcriptional regulation of GHR. Following transcription of the *GHR* gene, the fate of the resulting pre-mRNA/mRNA/protein is influenced by different processes which impact the functionality of the resulting protein product. The 3 arrows indicate the possible post-transcriptional modifications limiting functionality of the mature GHR protein (**1**) Alternative splicing of the pre-mRNA has been described, where exon 9 is partly or fully being skipped. This introduces premature stop codons, leading to formation of truncated GHR proteins lacking the entire intracellular domain. This renders the receptor functionally dominant negative when the truncated variants complexes with full length variants. Regulation of this alternative splicing is still incompletely understood; (**2**) Sparse reports have evaluated miRNA targeting of the GHR mRNA and identified miRNA (miR)-129-5p, miR-142-3p, miR-202, and miR-16 to target GHR in cell lines. The regulation and the significance of miRNAs in GHR regulation is still unknown; (**3**) TNF-α-converting enzyme (TACE) is upregulated during inflammation and functions as a “sheddase” for TNF-α but does also target the GHR protein. Cleavage of the GHR ecto-domain (indicated by scissor and arrow) disrupts GHR function and releases the ecto-domain as GH binding protein. TACE functions mainly in the secretory pathway but also on the cell membrane.

Alternative splicing of the GHR precursor mRNA can lead to synthesis of signaling incompetent GHR (Figure 4). The GHR gene is composed of 10 exons. Three major variants of the GHR mRNA have been reported in humans and are designated full length, truncated GHR 1–277, and truncated GHR 1–279, respectively. As stated above, these variants are formed as a consequence of alternative precursor mRNA splicing in exon nine leading to removal of 26 base pairs (variant 1–279) or the entire exon 9 (variant 1–277). Both cases lead to a translational frame shift that introduces a stop codon 3–4 amino acids after the GHR transmembrane domain, leading to truncation of the entire cytoplasmic domain. Reports suggest a dominant negative effect of these variants as they can dimerize with full length GHR which renders the dimer signaling incompetent [75]. The GHR variants were first identified in cell lines and later in human liver, muscle and fat. Although there were differences in the ratios of the GHR variants in these three tissues, the full length version of the GHR is the most abundant, whereas the 1–279 variant constitutes 4–10% and the 1–277 variant only less than 1% [75,76,77].

A homozygous mutation leading to synthesis of the 1–277 GHR variant has been reported and results in short stature due to the lack of GH-induced signaling. This conditions is known as Laron Syndrome (LS) [78]. Several other homozygous inactivating mutations in the GHR result in LS [79].

The regulation of the alternative precursor mRNA splicing is not known. A single study has looked at the adipose depots in lean and obese women and evaluated the level of full length GHR and the 1–279 variant and found a positive correlation of the variant with obesity in subcutaneous fat. Due to the dominant negative effect of the GHR variant, a decreased responsiveness to GH is expected with increasing obesity [35]. Whether inflammatory mediators influence alternative splicing is still unclear, but regulation of alternative splicing offers a potential mechanism for modulation of the responsiveness to GH.

Regulation of GHR translation might also be mediated by binding of miRNAs to the GHR mRNA in order to negatively impact translation or mRNA stability, although reports are sparse. One study has evaluated the effects of a series of miRNA identified in silico for their effects on human GHR expression in HEK293 cells and in two cancer lines (MCF7 and LNCaP) [80]. They conclude that miRNA (miR)-129-5p, miR-142-3p, miR-202, and miR-16 are targeting human GHR and inhibit its translation in the three cell lines. Evidence for miRNAs regulating GHR is emerging but its significance is still in question. At the same time, an understanding of its regulation and physiological impact in conditions like inflammation are also lacking.

As stated above, the level of functional surface exposed GHR is also affected by proteolytic shedding of the GHR ecto-domain. In mice, the GHBP is produced by alternative splicing of the GHR transcript [81], whereas GHBP in humans is derived from the GHR protein itself through enzymatic cleavage of the membrane bound receptor. The primary enzyme responsible for the ecto-domain shedding is TNF-α-converting enzyme (TACE) also named ADAM17, a metalloproteinase of the ADAM (A Disintegrin and Metalloproteinase Domain) family, while other metalloproteinases like ADAM10 might also induce the cleavage, although to a lesser extent [82,83]. TACE was identified as the protease cleaving membrane bound pro-TNF-α. It does, however, cleave a variety of membrane bound proteins, including adhesion molecules, EGF-receptor ligands and cytokine- and growth factor receptors including GHR. TACE is membrane bound and exerts its catalysis on target proteins in the Golgi, where TACE is primarily active, else the cleavage can occur on the cell surface [84,85]. TACE is ubiquitously expressed and its expression is increased during inflammatory conditions like rheumatoid arthritis and IBD, where increased TACE expression and protein levels are observed in the epithelial lining in both UC and CD [86,87,88,89]. Tissue inhibitor of metalloproteinases (TIMP)3 is an inhibitor of TACE and impacts the maturation process and activation of TACE through the secretory pathways and also limits its proteolysis in the extracellular environment [90]. Experimental colitis in the dextran sodium sulfate (DSS) treated mice showed increased TACE and decreased TIMP3 expression. Additionally, treatment with TACE inhibitors leads to lessened body weight loss, disease activity index, colonic myeloperoxidase activity as well as inflammatory cytokine levels [91]. An in vivo study of the C3H/HeJ mouse showed a blunted GH response in hepatocytes following lipopolysaccharide (LPS) stimulation due to resulting GHR proteolysis, a finding which was unrelated to changes in GHR mRNA levels [92].

Collectively, receptor abundance is influenced by inflammation in various ways. As a key parameter for GH responsiveness, inflammation-mediated effects on GHR expression and surface exposure can significantly influence GH sensitivity. The level of circulating GHBP is generally believed to reflect GHR status [93]. To our knowledge data on GHBP levels during inflammatory conditions and in particular IBD is lacking in human subjects. Validation of GHBP as an indirect measure of GHR status could therefore be of clinical interest for evaluating GH resistance.

### 3.3. Regulation of Intracellular Signaling during Inflammation

**Figure 5:**
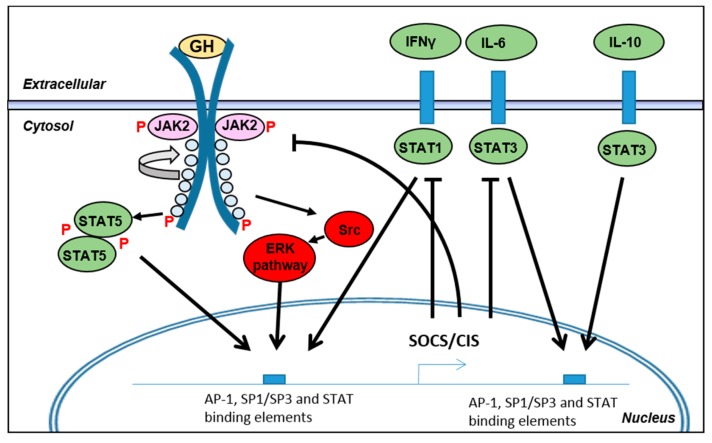
Regulation of GHR mediated signaling. During inflammation multiple cytokines elicit cellular signaling through their respective receptors. In order to regulate cytokine signaling negative feedback loops exist which includes the family of suppressor of cytokine signaling (SOCS) proteins including SOCS1–7 and CIS (cytokine-induced STAT inhibitor). The SOCS proteins target the receptors for proteolysis and limits phosphorylation of signaling intermediates. GHR signaling itself induces SOCS expression (mainly SOCS1 and 3) to terminate its own signaling. Several inflammatory mediators also induce SOCS expression which in turn creates an increased negative impact on the GHR signaling. IL-10 functions as an anti-inflammatory cytokine—note that IL-10 induces SOCS expression but is not itself inhibited by their expression whereas pro-inflammatory cytokine receptors are. IL-6 is the best described cytokine to negatively impact GHR signaling. GHR, accordingly, appears to be impacted by the endogenous regulatory systems in place to prevent excessive cytokine signaling. Arrows and blocked arrows indicate inducing and inhibiting actions, respectively, of the SOCS/CIS negative feedback loop on receptor signaling.

Inflammatory mediators may, besides affecting GHR expression, also interfere with intracellular GH-mediated signaling (Figure 5). Whereas TNF-α and IL-1β affected GHR expression, IL-6 has been shown to inhibit GHR action by inducing expression of members of the SOCS family. Regulation of receptor tyrosine kinases (RTKs), including GHR, is important for controlling cellular signaling. Feedback loops exist to curtail their activity to prevent excessive activity and to reduce the risk of oncogenic development.

The SOCS protein family consists of 8 members (SOCS1–7 and CIS (cytokine-induced STAT inhibitor)). A common feature of the family is the presence of an SH2 domain and a SOCS-box domain. Through the SH2 domain, SOCS binds to the phospho-tyrosine of activated RTKs and competitively inhibit binding of docking proteins. The SOCS-box domain recruits Cullin 5/2 to form the Cullin-Ring E3 ligase complex. The assembly of this complex ultimately leads to ubiquitination of the RTK. Ubiquitination of the RTK induces lysosomal targeting and degradation both in the case of mono- and poly-ubiquitination in order to terminate the signaling [94,95]. SOCS1 and SOCS3 contain an additional KIR motif which has a kinase inhibitory function, thereby limiting further phosphorylation of docking proteins [95]. The SOCSs have preferences for different RTKs and the SOCS proteins may also be expressed in a cell-specific manner which increases the complexity of their regulatory functions due to spatiotemporal regulation [96].

SOCS1, SOCS2 and SOCS3 are generally the best characterized members where ablation leads to dramatic phenotypes. *SOCS1*^−/−^ mice are hypersensitive to interferon (IFN)-γ [97], *SOCS2*^−/−^ show increased growth due to defects in regulation of the GH-IGF-1 axis [98], and *SOCS3*^−/−^ mice are embryonic lethal [99]. The emerging knowledge and the basic properties of the SOCS proteins have been reviewed elsewhere [95,100].

Expression of the SOCSs proteins are regulated by RTK activity to providing a negative feedback loop on cytokine signaling. Studies have shown that GHR itself induces expression of primarily SOCS3, but also of SOCS1, SOCS2 and CIS in 3T3 fibroblasts [101] as well as in different rodent tissues [96,102]. Additionally, overexpression of SOCS1 and SOCS3 is capable of blocking STAT5b activation while an intermediate effect was observed for SOCS2 and CIS in COS-1 cells [103].

The mechanism regulating this negative feedback on the RTKs and on GHR in particular is mediated by the activated STAT transcription factors. It has been shown that STAT5b is involved in induction of SOCS1-3 and CIS transcription in mouse liver [96,103]. Studies in HUVEC cells suggest that expression of SOCS3 is regulated by the simultaneous binding of AP-1, SP1/SP3 and STAT transcription factors to conserved binding-elements at its promoter/enhancer [104]. Increased SOCS3 expression has also been reported in fasted rats as part of the GH resistance [39].

Besides the presence of a GH-induced negative feedback loop through SOCS expression, a number of cytokines also induce expression of SOCS proteins. SOCS1-3 and CIS are expressed in low levels in most cells and can be induced pronouncedly by IFN-γ (SOCS1), IL-6 and IL-10 (SOCS3) [105,106,107]. Note that SOCS3 does not inhibit the IL-10 receptor itself and therefore IL-10 acts as an anti-inflammatory mediator for other cytokine receptors [107]. Whether LPS induces SOCS expression directly is not clear, but indirect induction through induction of the aforementioned cytokines leads to SOCS induction.

In relation to systemic inflammation, IL-6 shows a major impact on GHR mediated signaling. Following binding of IL-6 to the IL-6 receptor, gp130 is recruited and results in a hexameric complex consisting of IL-6, IL6R and gp130. Subsequent intracellular signaling leads to STAT3 activation and expression of target genes including acute phase reactants like CRP and serum amyloid protein A but also inducing SOCS1 and SOCS3 [108,109,110]. SOCS3 targets the activated gp130 receptor to inhibit the IL-6 receptor complex signaling but at the same time SOCS 1 and SOCS3 have been reported to target GHR as well [111]. In vitro, IL-6 induces SOCS3 expression in Huh-7 cells, which limits the GH-induced IGF-1 response. In vivo, one dose of LPS (2.5 µg/g body weight) in the peritoneum leads to reduced liver IGF-1 expression and increased SOCS3 expression after 5 h. The increased SOCS3 expression could partly be reverted by a neutralizing IL-6 antibody administered 1 h post LPS dosing, whereby IGF-1 expression was restored to normal [65]. In mouse hepatocytes, IL-6 inhibited GH-induced IGF-1 and Spi 2.1 expression as a consequence of reduced nuclear STAT5 DNA binding, which points to a role for the SOCS proteins in GH action [112]. Both systemic and local intestinal levels of IL-6 are elevated in patients with active IBD. Targeting the IL-6 receptor in vivo has shown a beneficial effect on experimental colitis and also a positive effect in human CD patients and in other inflammatory conditions, notably rheumatoid arthritis [110,113,114]. Thus, the chronic inflammatory state and elevated cytokine levels results in increased SOCS expression to limit cytokine signaling and, consequently, GHR signaling is affected as well.

Studies of experimental colitis in rodents have shown that SOCS1 and SOCS3 levels are increased during inflammation and that both the heterozygous state and ablation of *SOCS1* induces more severe intestinal inflammation [115,116,117]. In IBD patients, expression of SOCS1 and SOCS3 has been reported to be upregulated during active disease [117,118,119,120,121,122].

These data jointly indicate that the SOCS proteins are important during inflammation for limiting cytokine-induced cellular signaling and thereby limit the inflammatory response. The simultaneous negative impact on GHR signaling seems to be a consequence of this.

Collectively, the mechanisms underlying inflammation-induced GH resistance comprise direct impacts on the GHR receptor itself through changes in transcription and translation, but also impacts the downstream signaling intermediates which targets GHR as well as inflammatory cytokine receptors.

## 4. Effects of Growth Hormone in the Intestine during Inflammation

### 4.1. Effects of GH on Mucosal Integrity

Disruption of the mucosal barrier integrity is central to the development of IBD, and mucosal healing is considered a hallmark for successful treatment. Because of this, it is important to understand the effects of GH on the intestinal barrier to get a clear understanding of the relationship between GH and IBD. It has been reported by Gilbert S. et al., that GH, through inhibition of NF-κB activation [71], decreases gut inflammation and improves or maintains gut barrier function, which ultimately inhibit development of IBD as shown in intestinal epithelial cell-specific STAT5 knockout mice [123].

The pro-barrier effects of GH are exhibited in multiple animals. In STAT5 knockout mice, which have much of their GH action blocked, intestinal barrier function is impaired [124], indicating that GH is necessary for maintaining gut barrier homeostasis. GH administration has also been claimed to improve gut barrier function and to limit bacterial translocation in rats [125] and humans [126]. The results of these studies solidify the positive correlation between GH action and intestinal barrier function.

### 4.2. Effects of GH on Immune Cells

GH also modulates the immune system through direct effects on immune cells. Most immune cells express GHR, with the highest expression in B cells, the lowest expression in T cells, and intermediate expression in other white blood cells. Within the subset of T cells, GHR expression is the lowest in cluster of differentiation 4+ T cells. Not only do immune cells express GHR, indicating that they may respond to GH, many immune cells also express GH [127]. In agreement with the pattern of GHR expression, *GH* gene and protein expression is highest in B cells, although the level of secreted GH was near the detection limit. The expression of both GH and GHR by B cells indicates that GH action in these cells may be through an autocrine/paracrine pathway. The authors did not find any correlation between B cell GH/GHR expression and serum Immunoglobulin G or IGF-1, respectively [127].

The effects of GH on immune cells are diverse. Despite GHR expression being low in T cells, increased GH acts in the thymus to increase T cell proliferation and development [128]. As GHR expression is highest in B cells, GH exerts some of its strongest immune effects on these cells. GH stimulates immunoglobulin production by B cells in an IGF-1-independent manner, as well as increasing B cell differentiation [129]. GH action also has effects in non-lymphocytes of the immune system, stimulating oxygen radical production by monocytes and neutrophils, as well as promoting neutrophil adhesion and attracting monocytes [130].

A macrophage-specific *GHR* knockout mouse showed that loss of GH action resulted in increased inflammation in the adipose tissue, further reinforcing the anti-inflammatory effect of GH seen in the intestines of IL-10 knockout mice [131].

These studies indicate that GH under some conditions may impact and regulate immune functions in order to limit inflammation. Together with the potential positive impact of GH on barrier function, GH might function to inhibit the inflammatory burden during IBD, although the effects are likely blunted by inflammation-induced GH resistance. The results from a cross over study of twelve healthy controls being dosed with GH for 3 weeks followed by a GH antagonist (pegvisomant) for 3 weeks showed some interesting and partly unexplainable results; here GH seems to mediate a slight increase in TNF-α and decrease in CRP and orosomucoid levels compared to pegvisomant treated individuals [132]. Conclusively, more studies are needed in order to evaluate the roles of GH in the complex setting of systemic inflammation before conclusions can be drawn.

### 4.3. Effects of GH in Experimental Models of Colitis and Short Bowel Syndrome (SBS)

A series of studies have evaluated the effects of GH in different animal models of experimental colitis. In the spontaneous colitis model using *IL-10* knockout mice, GH showed a positive effect on colon histology and significantly reduced epithelial apoptosis possibly by increasing expression of the anti-apoptotic BCL-2. It simultaneously increased epithelial proliferation. Increased apoptosis of mononuclear cells in the lamina propria in vivo was also reported along with a negative impact on IL-6 signaling in T84 and Jurkat cells [133]. Later, the same group reported that *IL-10* knockout mice were less responsive to GH, an effect which was alleviated by either neutralisation of Tκ-α or by chronic administration of GH. GH also reduced NF-κB activation in colitis, which is a potential anti-inflammatory effect of chronic GH administration in IBD. Neutralisation of TNF-α has been shown to upregulate colonic epithelial GHR in the *IL-10* knockout mice [71]. In another study, using 2,4,6-trinitro-benzenesulfonic acid (TNBS)-induced colitis, it was shown that STAT5b is important for maintaining barrier integrity by increasing epithelial cell survival hence indicating that GH positively affects mucosal healing during intestinal inflammation [124]. In a similar manner, GH affects survival, induction of remission, and mucosal repair in DSS-induced murine colitis, which was shown in a transgenic mouse line expressing the bovine *GH* gene. Although high GH did not alter susceptibility to DSS-induced colitis, survival, rate of remission, and mucosal repair were improved. These kind of studies are, however, hard to evaluate as the transgenic mice are different from baseline, including differences in body size/weight/composition, drinking habits and intestinal weight/length [134]. TNBS-induced colitis in rats also shows beneficial effects of GH on histology measures as well as reduction in myeloperoxidase activity [135,136]. In another study, GH was shown to have no effect on inflammation in a rat peptidoglycan-polysaccharide-induced colitis model [137]. Lastly GH treatment of septic rats limited formation of intestinal mucosal injuries as well as bacterial translocation [138].

### 4.4. Clinical Evaluation of GH in Inflammatory Bowel Diseases (IBD) Patients

Patients with active IBD generally have normal levels of GH along with a reduced level of IGF-1 and some of the IGF-1 binding proteins. The biochemical image points to the presence of GH resistance in this group of patients which is most likely mediated by the ongoing inflammatory process as IGF-1 levels rise following induction of disease remission [139,140,141,142,143,144,145,146]. This notion is further underlined by the finding that these patients have elevated levels of pro-inflammatory cytokines both systemically and locally in the tissue [11,147,148].

In children, a series of smaller trials have been performed to evaluate the effects of GH on primarily growth retardation [149,150,151,152,153,154,155,156]. Most of these studies showed an effect on growth velocity, where some failed to do so. Additionally, the studies failed to/did not aim at showing an effect of GH on disease severity or the induction of remission.

In adults, Slonim et al. [157] evaluated the effects of GH in a cohort of CD patients focusing on disease severity. Overall they reported a significant decrease in the Crohn’s disease activity index (CDAI) score in the GH treated patients after having received subcutaneous GH injections for 4 months compared to placebo. Patients treated with GH also showed a decreased need for concomitant medication (prednisone and immunosuppressants) relative to baseline compared to the placebo group. The positive results of GH-treatment in CD patients have not been replicated since in external patient cohorts.

Besides active IBD, GH has also been utilized for treating SBS, a complication which might occur among IBD patients following surgery and bowel resection. The goal when treating SBS patients is to improve enteral autonomy in order to limit requirement for parenteral nutrition and intravenous fluids. A study in rabbits has shown that, after small bowel resection, GH and epidermal growth factor increased nutrient uptake and also increased microvillus height (which increases surface area and absorptive capacity [158]. In humans, Byrne et al. [159] have shown that four weeks treatment of SBS patients with GH and glutamine positively aided intestinal compensation along with reducing requirement for parenteral nutrition, an effect which superseded glutamine supplementation alone and an effect that was maintained even after ending GH supplementation. Additional studies show similar effects of GH supplementation in SBS patients and show positive effects on absorption, protein synthesis and glutamine availability [160,161]. Initial focus on the potential nutritional and metabolic consequences of these patients is important for a successful surgical outcome. Laurberg S. and coworkers did a series of studies on UC patients undergoing ileoanal J-pouch surgery (removal of the colon) and evaluated the effects of GH supplementation twice daily 2 days pre- until 7 days post-operatively. In the study cohort of 24 patients they found that GH shot term prevented weight loss (placebo lost 4.3 ± 0.6 kg) and enhanced fluid balance significantly compared to placebo. The effect of GH on lean tissue mass was maintained over a period of 90 days and GH additionally improved the recovery of perioperative muscle strength and fatigue [162,163,164].

## 5. Discussion and Conclusions

It is clear that inflammation per se impacts the ability of GH to mediate its anabolic functions. Multiple mechanisms appear to influence GH responsiveness, including (1) metabolic status; (2) steroid treatment; (3) inflammation-mediated repression of GHR expression (primarily reported effect of TNF-α and IL-1β); (4) induction of negative regulators of cytokine signaling, e.g., the SOCS proteins (reported effect of IL-6); (5) proteolytic cleavage of surface GHR (effect of TACE and related proteases); along with less understood mechanisms including (6) regulation of alternative splicing of the GHR precursor mRNA; and (7) miRNAs. Accordingly, an ongoing inflammatory process impacts formation of GH resistance in several ways and, therefore, may generate a significant impact on GH’s functions.

Based on the studies presented in this review, the effects of GH in treating active IBD appear to relate mostly to improvement of the metabolic status of the patients. This assumption is based on the finding that GH seems to increase protein synthesis, enhance intestinal absorption and function to mediate a more anabolic state leading to increased lean body mass. Although smaller studies conducted in cells and animals points to potential positive effects related to barrier function and effects on immune cells, the understanding of these functions in the actual disease is not clear. The direct effects of GH on the ongoing inflammatory process in patients with IBD appear indirect since no reports are available relating to direct anti-inflammatory effects of GH.

As inflammation negatively impacts GHR functions and consequently lead to impaired IGF-1 levels, although GH levels are not reduced, the primary “inducer” in these patients must accordingly be the underlying inflammatory process. Thus, we propose that inflammation ultimately down regulates GH action via effect on the GHR or GH-induced intracellular signaling molecules and that targeting the inflammatory process is key for effectively treating these patients.
